# A Simple Remedy for Cramps in the Lower Extremities

**Published:** 1847-07

**Authors:** S. A. Bardsley

**Affiliations:** Manchester


*A simple remedy for Cramps in the lower extremities.* By Dr. S. A. Bardsley, Manchester.—Having myself been for many years a martyr almost every night to this torturing malady, and having tried in vain many of the "thousand and one" remedies usually prescribed for relief, I was at length led to reflect upon a fact which had hitherto escaped my attention, viz., while sleeping in a chair, with my lower limbs, if not touching the floor, yet so depending as to form an inclined plane with the whole of my frame, that I was in this position never disturbed by cramps; and upon inquiry I found other sufferers from habitual cramps were under the same predicament. These facts, in connection with some physiological considerations, induced me to put into practice the following plan, which has proved decidedly successful. My plan is to sleep upon an inclined plane, which is effected by taking care that the bed or mattress should incline twelve inches from the upper to the lower part of the bed; and for this purporpose the lower feet were cut down so as to form this inclination. I will now state two facts, which are sufficient tests that neither the imagination nor intemperate diet were the causes of my habitual cramps. 1st. That after my trial of the inclined plane for seven consecutive nights with complete success, the housemaid, unknown to me, had raised my bed to its usual horizontal level, and, unconscious of the change, I went to sleep, when shortly afterwards the cramps were so severe as to compel me twice to alarm the family by my cries and moans; and it was not until I arose in the morning that I discovered the change in the form of my bed. 2d. The other test is the one which I made six weeks ago. After very spare diet of twenty-four hours, I replaced my bed from the inclined to an horizontal position, when, shortly after, I awoke with dreadful cramps—so violent in the muscles of the thigh and legs as to require two persons to hold the limbs down in order to apply friction, with stimulants, both external and internal; indeed, the paroxysm was so severe and continued as to be accompanied with sickness and faintness. I deem it necessary to give a caution to sufferers from cramps, that the disorder is almost always connected with a weak or imperfect state of the digestive organs, and therefore, although the method now stated for relief will allow the sufferer several luxuries hitherto forbidden, yet there must be limits placed to such indulgences if he expects to pass the nights entirely free from his malady.—*Ibid.*

